# Differential features of early childhood motor skill development and working memory processing: evidence from fNIRS

**DOI:** 10.3389/fnbeh.2023.1279648

**Published:** 2023-09-26

**Authors:** Quanliang Zheng, Aiping Chi, Bing Shi, Yimin Wang, Qing Ma, Fang Zhou, Xianmei Guo, Menghan Zhou, Bowei Lin, Ke Ning

**Affiliations:** ^1^School of Physical Education, Xi'an Fanyi University, Xi’an, China; ^2^School of Physical Education, Shaanxi Normal University, Xi’an, China; ^3^The First Kindergarten of Xi'an Gaoxin, Xi’an, China; ^4^Xi'an High-Tech Zone 22nd Kindergarten, Xi’an, China

**Keywords:** early childhood, motor skill, working memory, functional near-infrared spectroscopy, oxygenated hemoglobin, prefrontal cortex

## Abstract

**Objective:**

The study investigated the differential characteristics associated with motor skill development and working memory processing during early childhood, thereby providing insights for understanding motor learning and cognitive development in young children.

**Methods:**

In total, 101 preschool children (age: 4–6 years) were recruited for this study. The motor skill development level and the working memory capacity of the children were assessed using the MOBAK Motor Development Assessment Scale and a block task paradigm, respectively. Functional near-infrared spectroscopy brain imaging technology was used to monitor hemodynamic signals in the prefrontal cortex (PFC) of the children while they completed different memory tasks. MATLAB software and the Homer2 plugin were used to calculate the oxygenated hemoglobin (Oxy-Hb) concentration in relevant brain regions during the tasks.

**Results:**

(1) The low motor skill group exhibited significantly lower accuracy during the three-memory load condition than during the two-memory load condition. Under both two-memory and three-memory load conditions, the high motor skill group exhibited significantly higher accuracy than the low motor skill group. (2) Significant differences in the Oxy-Hb concentration were observed in the left dorsolateral PFC (L-DLPFC), and right and left triangular part of the Broca’s area (R-PTBA and L-PTBA, respectively) between the two memory difficulty levels for the high motor skill group. The Oxy-Hb concentration was significantly higher during the three-memory load condition than during the two-memory load condition. Under the two-memory load condition, the high motor skill group exhibited significantly higher Oxy-Hb concentration in the L-DLPFC and L-PTBA regions than in the low motor skill group. Under the three-memory load condition, the high motor skill group exhibited significantly higher Oxy-Hb concentration in the L-DLPFC, R-PTBA, and L-PTBA regions than the low motor skill group.

**Conclusion:**

A close association was observed between the motor skill levels and working memory in young children, with higher motor skill levels being associated with more pronounced brain activation patterns during working memory tasks.

## 1. Introduction

Motor skills are considered a fundamental pattern of non-natural motor learning (Barnett et al., 2016). They form the basis for individuals’ physical activities and interactions with the external environment (Gottwald et al., 2016). According to different action types, basic motor skills can be categorized as mobility, manipulative, and balance skills. Motor areas (such as the primary motor cortex) and more widely distributed non-motor areas [such as the prefrontal cortex (PFC)] form the neural foundation of motor behavior (Diamond, 2000; Wu et al., 2021). These areas collaborate and function under the coordination of the central motor system to execute movements (Wade and Whiting, 1986; Wu et al., 2021). The PFC plays a role in various cognitive processes and top-down regulation of motor learning (Chambon et al., 2011), networking with the basal ganglia and guiding behavior in complex cognitive and motor tasks (Diamond, 2000). Motor learning-related studies have shown that the oxygenated hemoglobin (Oxy-Hb) response in the PFC is a sensitive indicator of motor learning (Ono et al., 2015). The co-activation between the PFC and the primary motor cortex develops continuously and increases with training enhancement (Wu et al., 2022). This is related to the involvement of the PFC in motor sequence learning (Polskaia et al., 2023). The acquisition of environmental target visual search and spatial action sequences relies on neural circuits forming the PFC. Actions are executed depending on the decision-making and selection processes of the PFC (Yang and Wang, 2018). Therefore, the PFC is closely related to motor learning and development (Wu et al., 2022).

Working memory is a core executive function involving the short-term storage, retention, and manipulation of perceptual information (Lou et al., 2019). Working memory is the foundation for higher levels of cognitive activities such as calculation, reasoning, and comprehension and is crucial in various cognitive domains including planning, skill learning, and problem-solving (Li et al., 2023). A close relationship has been reported between working memory and children’s academic achievement and cognitive development (Blankenship et al., 2018; Quilez-Robres et al., 2021). The PFC is a key player in higher levels of cognitive activities, including working memory (Narayanan et al., 2005; Jia et al., 2022; Voegtle et al., 2022; Li et al., 2023). Memory training can enhance prefrontal neuron activity and strengthen the connectivity between the PFC and parietal cortex (Constantinidis and Klingberg, 2016). Therefore, working memory and motor skill acquisition share a common neural basis within the prefrontal region (Ludyga et al., 2018). Working memory serves as the foundation for several behaviors, from perception to higher-order cognition and behavioral control (Ludyga et al., 2018). Visuospatial working memory is considered central for action perception and action sequence learning (Bo and Seidler, 2009; Seidler et al., 2012; Buszard et al., 2016; Jongbloed-Pereboom et al., 2019). The memory and encoding of actions rely on cognitive processes involving working memory (Bo et al., 2011; Wozniak et al., 2022), and action execution requires the engagement of executive functions involving working memory (Yang and Wang, 2018). The visuospatial memory capacity is associated with action execution strategies (Duijn et al., 2017). Consequently, the motor skill proficiency and working memory capacity seem to be linked, with the PFC playing a substantial role in both phenomena.

Early childhood is a critical period for both motor and cognitive development (Stodden et al., 2008; Xiongtao et al., 2019). When the body and brain develop naturally, young children are equipped with basic motor abilities, and early motor development is an external manifestation of cognitive development. Motor skill development in early childhood requires support from higher levels of cognition (Raichlen and Alexander, 2017). Furthermore, when motor development in early childhood is optimal, it contributes to the continuous advancement and complexity of cognitive development in children (Wu et al., 2021; Lin et al., 2023). The plasticity of brain cortical regions provides a neural foundation for achieving higher levels of motor and cognitive development during this stage (Huttenlocher, 2002; Hilderley et al., 2023). In an intervention study, 8 weeks of coordinated tennis training enhanced motor abilities in young children and increased oxygen concentration in the PFC during working memory tasks (Lai et al., 2020). This may be related to the activation of PFC neurons during motor learning (Anguera et al., 2010). The improved neural efficiency of working memory offers a learning and execution foundation for motor control. Based on these findings, this study explored whether differences in working memory abilities exist between young children with high and low motor skills. Additionally, the study determined the specific regions of the PFC that exhibit differences during working memory tasks in young children with different motor ability levels.

As cognitive neuroscience has advanced, functional near-infrared spectroscopy (fNIRS) technology has been extensively used in cognitive neuroscience research (Piazza et al., 2019), which has resulted in significant progress and breakthroughs in areas such as executive functions and brain functional connectivity in children (Xie et al., 2021). fNIRS is a non-invasive optical imaging technique that can be used for measuring changes in Oxy-Hb and deoxygenated hemoglobin (Deoxy-Hb) concentrations, which are related to brain function activity. During fNIRS, participants have to wear a cap embedded with near-infrared detector–emitter pairs. Each pair creates a channel, allowing fNIRS to provide better neural signal localization compared with EEG. Additionally, fNIRS is associated with advantages such as portability, relatively high temporal and spatial resolution, ecological validity, minimal participant restrictions, and applicability to diverse populations (Carruthers, 2013; Piazza et al., 2019). These benefits offered make fNIRS particularly suitable for studying cognitive neuroscience in young children. Therefore, fNIRS was employed here to explore the relationship between motor development and working memory in young children.

## 2. Materials and methods

### 2.1. Participants

The study participants were children (age: 4–6 years) recruited from two kindergartens in Xi’an. Children who were right-handed and had normal or corrected vision were included in the study. Children with psychiatric disorders or currently receiving medications were excluded. The guardians were informed about the research project. When they voluntarily agreed to participate, they signed informed consent forms. In total, 114 children were recruited for the study. During the formal testing period, five children could not participate because of H1N1 infection; three children could not understand the rules of the working memory test and hence were not tested; and two children were too active and could not remain calm during the test, and thus, they did not complete the working memory test. Finally, 104 children completed all the test items. After the near-infrared data were processed, three children were excluded from the study because their near-infrared data were of poor quality (excessive artifacts). Ultimately, data of 101 children were included in the analysis. The 101 children included 48 boys and 53 girls whose mean age was 60.53 ± 3.28 months. This study was approved by the Ethics Committee of Shaanxi Normal University (202316006).

### 2.2. Working memory test

#### 2.2.1. Test materials and design

**Figure 1:**
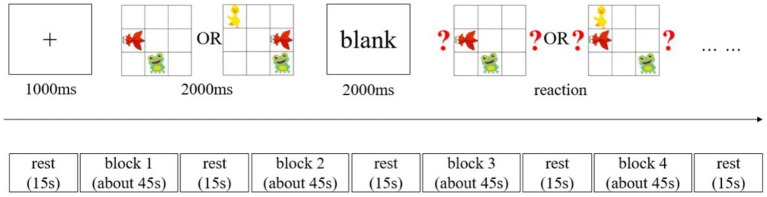
Workflow of the working memory test.

The visuospatial working memory of young children was tested using a block design. The test task was based on established research paradigms of visuospatial working memory in children (Simmering, 2012; McKay et al., 2021), with moderate modifications. The stimuli presented were three cartoon images of a fish, a frog, and a duck randomly placed within a nine-square grid (Figure 1). Only the fish and frog were present for the two levels of visuospatial working memory load. All three animals were included for the three levels of load. During the experiment, the animals’ spatial positions were presented in different locations each time, and they were not presented adjacent to each other to avoid any learning effects.

All memory task presentations and data collection of behavioral measures were conducted using E-prime 3.0 software (Psychology Software Tools Inc., Sharpsburg, PA, United States). Figure 1 presents the task presentation. The memory tests consisted of two memory load conditions: a two-memory load and a three-memory load. In each trial, a fixation point was first presented at the screen’s center for 1,000 ms, followed by the stimulus picture presented for 2,000 ms. This was first followed by a blank screen period of 2,000 ms and then a picture containing a question mark. This required the participants to judge whether a change had occurred in both the animal and position compared to the stimulus picture. The participants had a fixed time to respond. While responding, they had to use the “D” key if the animal and position were the same as the stimulus picture or the “J” key if they were different. This constituted one trial. Each block included six trials, and the blocks presentation were presented in the following order: two blocks with two-load conditions, followed by two blocks with three-load conditions. A 15-s rest period was maintained between each block. Behavioral data (reaction time and accuracy) and hemodynamic data from the brain regions of interest (ROI) were collected during the formal experiment. Before the start of the formal experiment, all participants received instructions and training. Before the first block of the formal test, they also had to complete a practice session. Consisting of four trials, including combinations of two-load and three-load visuospatial memory tasks. After the practice session, a 15-s rest period was maintained before the formal testing began.

During the experiment, all stimuli were presented on a 15.3-in computer display. The stimulus materials were centered and occupied 75% of the screen, which had a white background color. The participants were made to sit at a distance of approximately 60 cm from the computer display.

#### 2.2.2. fNIRS data acquisition

**Figure 2:**
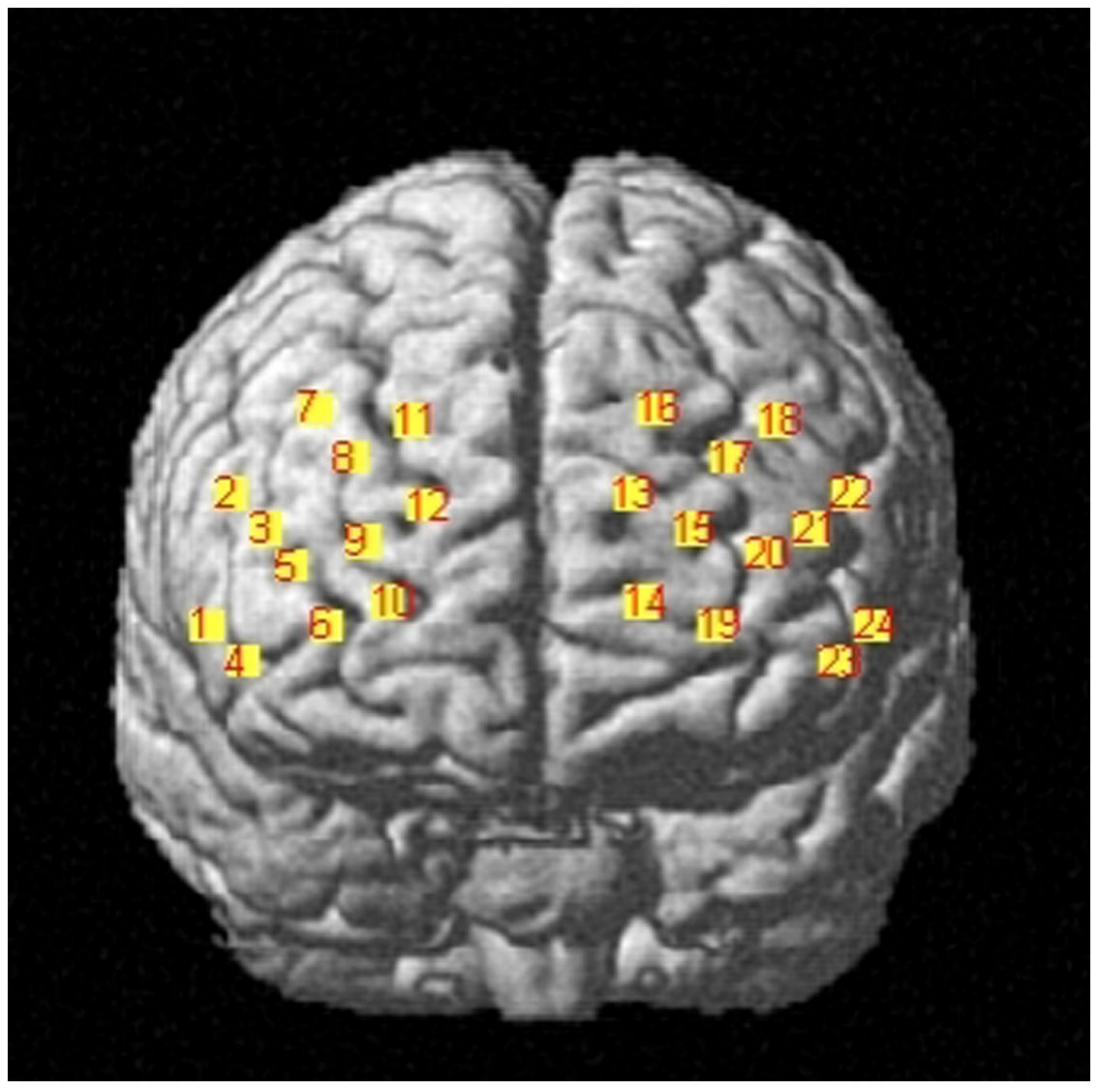
Brain division map of each channel.

The Artinis portable near-infrared brain imaging system (brite MKIII, Artinis Corporation, Holland) was used to monitor PFC hemodynamic signals of the participants while they were executing the working memory tasks. The OxySoft data acquisition and analysis software was used for synchronized data collection. In this study, the equipment included 10 emitting and eight receiving optodes, respectively. Based on the distribution characteristics of working memory-related brain regions and considering the arrangement used by previous studies for ROI (Xiang et al., 2022), a 2 × 12 channel layout was adopted here, resulting in 24 collection channels distributed over the PFC (Figure 2). The distance between the emitting and receiving optodes was approximately 3 cm. The light sources emitted at 760 and 850 nm, while the sampling frequency was set to 25 Hz.

To obtain three-dimensional coordinate localization for each channel position, we used the three-dimensional head position modeling system (Patriot, Polhemus, United States). Spatial registration was performed using the NIRS_SPM software package in Matlab. Based on the specific distribution of the 24 channels, six ROI were defined. Table 1 presents the corresponding channel-to-brain region mapping.

**Table 1:** Each channel corresponds to the location information of the brain region of interest.

Frontal cortex region	Channel
Right dorsolateral prefrontal cortex, R-DLPFC	3, 4, 5, 7, 8, and 11
Left dorsolateral prefrontal cortex, L-DLPFC	16, 17, 18, 20, 21, and 23
Right triangular part of the Broca’s area, R-PTBA	1, 2
Left triangular part of the Broca’s area, L-PTBA	22, 24
Right frontopolar area, R-FPA	6, 9, 10, and 12
Left frontopolar area, L-FPA	13, 14, 15, and 19

### 2.3. Motor skills testing

The “MOBAK-KG” motor skill assessment scale, developed by Herrmann et al. (2018), is used in young children. The scale consists of two parts: manipulative and locomotor skills (Herrmann et al., 2018). Following are the tests included in the manipulative skills part of the scale: single-handed throwing at a fixed target, two-handed catching a bouncing ball, two-handed clapping catch, and dribbling with feet around obstacles. The tests included in the locomotor skills part are as follows: walking forward and backward on a balance beam, forward rolls on an incline, one-legged hopping back and forth, and running forward and backward. Each skill is evaluated using 3–5 criteria. The evaluator scores based on the number of successful attempts according to the criteria. If the skill is not completed, a score of “0” is assigned. Successful skill completion corresponds to a specific score. Each test item on the scale is scored on a scale of “0–2” points. The scoring range for locomotor and manipulative skills is “0–8” points each, with the overall scoring range for the scale being “0–16” points.

The MOBAK scale has been extensively used to examine motor skills and cognitive development in children (Ludyga et al., 2018, 2020). Because this scale has no specific criteria for classifying high and low scores, this study used the median split method to classify the scores. Children with scores ranging from 1 to 8 were assigned to the low group, while those with scores ranging from 9 to 16 were allocated to the high group (Ludyga et al., 2018). The median split method has been previously validated as a grouping technique in studies and is not expected to lead to significant bias in data analysis results (Iacobucci et al., 2015).

### 2.4. Data processing and statistics

#### 2.4.1. Data preprocessing

All behavioral data of the participants were processed, merged, and organized using the E-Merge3 function in e-prime 3.0 software. The merged data were imported into Excel for further data preprocessing according to previous studies (Yifan et al., 2018). Data preprocessing involved removing reaction times with average values exceeding ±3 standard deviations.

The fNIRS data were preprocessed using MATLAB software (R2013b, MathWorks Corporation, United States). The Artinis company plugin package, oxysoft2matlab, was used to convert the raw data files (*.oxy5 and *.oxyproj) into the *.nirs format. The intensity data were converted to Oxy-Hb and Deoxy-Hb concentration data using the Homer2 plugin package. Motion artifacts were corrected for all channels, with the flagging of bad channels. Respiratory and cardiac components >0.1 and < 0.01 Hz were removed using a bandpass filter. For task-related data, a baseline correction was performed using the 2-s data preceding the start of each block. Based on the task design, each block was expected to take approximately 45 s to complete. Therefore, data from 2 to 45 s after the onset of the same type of stimuli were averaged together. Finally, mean values of Oxy-Hb, Deoxy-Hb, and total hemoglobin concentrations were obtained for both working memory tasks. Oxy-Hb has a higher signal-to-noise ratio and greater sensitivity to changes in brain oxygenation (Strangman et al., 2002; Lindenberger et al., 2009). Therefore, in the subsequent analysis, Oxy-Hb was chosen as the indicator for analyzing blood oxygen concentration. The NIRS_KIT software package was used to process the activation of the blood oxygen concentration during the task.

#### 2.4.2. Statistical analysis

Data were statistically analyzed using SPSS 25.0 software (IBM Inc., Chicago, IL, United States). Pearson’s correlation analysis was conducted to examine the correlations between motor skill scores and behavioral outcomes during the working memory period and between motor skill scores and Oxy-Hb concentration results. A 2 × 2 mixed-design ANOVA was conducted to test for interaction and main effects of different participant groups on behavioral outcomes and Oxy-Hb concentrations during the two working memory conditions. Based on the analysis results, simple effects testing and paired comparisons were conducted. Descriptive statistics were reported as mean ± standard deviation, with a significance level of α = 0.05.

## 3. Results

### 3.1. Results of correlation analysis

A correlation analysis was conducted to examine the relationship between motor skill scores and behavioral outcomes, and between motor skill scores and Oxy-Hb concentration results during the working memory period (Table 2). Motor skill scores were significantly and positively correlated with Oxy-Hb data in various ROI under both working memory conditions, and the correlation coefficients were 0.2–0.6. Motor skill scores were also significantly and positively correlated with behavioral accuracy under both working memory conditions, and the correlation coefficients were 0.2–0.4. However, no significant correlation was observed between motor skill scores and behavioral reaction time under either working memory condition.

**Table 2:** The correlation between motor skills and working memory indicators.

Indicators of working memory tests		Correlation coefficient (*r*)	
		Two-memory load condition	Three-memory load condition
Oxy-Hb concentration (*mol/L*)	R-DLPFC	0.358^**^	0.430^**^
	L-DLPFC	0.292^**^	0.508^**^
	R-PTBA	0.239^**^	0.489^**^
	L-PTBA	0.359^**^	0.507^**^
	R-FPA	0.290^**^	0.356^**^
	L-FPA	0.203^*^	0.388^**^
Behavioral outcomes	Reaction time (*ms*)	−0.031	−0.029
	Correctness rate (*%*)	0.201^*^	0.391^**^

^*^p < 0.05, ^**^p < 0.01.

### 3.2. Behavioral results

The median split method was used to divide the participants into high and low-motor skill groups. A 2 (Motor Skill: High, Low) × 2 (Memory Difficulty: 2 Memory Load, 3 Memory Load) mixed-factor ANOVA was conducted to statistically analyze the behavioral outcomes of different groups under different working memory task loads (Table 3).

**Table 3:** Behavioral statistics results for different motor skills in working memory tasks (M ± SD).

Variable	Low motor skill group		High motor skill group	
	(*N* = 23 males/23 females)		(*N* = 25 males/30 females)	
	Two-memory load condition	Three-memory load condition	Two-memory load condition	Three-memory load condition
Reaction time (ms)	2542.32 ± 784.78	2631.50 ± 921.01	2418.32 ± 677.38	2571.13 ± 1005.94
Correctness rate (%)	0.68 ± 0.17	0.63 ± 0.15	0.78 ± 0.16	0.78 ± 0.16

The reaction time results revealed no significant interaction effect between motor skills and memory difficulty [*F*_(1, 99)_ = 0.115, *p* = 0.735, *η^2^* = 0.001]. The main effects of motor skills and memory difficulty were not significant [*F*_(1, 99)_ = 0.414, *p* = 0.521, *η^2^* = 0.004], and [*F*_(1, 99)_ = 1.668, *p* = 0.199, *η^2^* = 0.017], respectively.

According to the accuracy results, a significant interaction effect was observed between motor skills and memory difficulty [*F*_(1, 99)_ = 4.739, *p* = 0.032, *η^2^* = 0.046]. The simple effects analysis evealed that for the low motor skill group, a significant main effect of memory difficulty was observed [*F*_(1, 99)_ = 7.371, *p* = 0.008, *η^2^* = 0.069]. The accuracy was significantly lower for the three-memory load condition than for the two-memory load condition. Under the two-memory load condition, the main effect of motor skills was significant [*F*_(1, 99)_ = 9.016, *p* = 0.003, *η^2^* = 0.083], with the high motor skill group exhibiting significantly higher accuracy than the low motor skill group. A significant main effect of motor skills was also observed under the 3-memory load condition [*F*_(1, 99)_ = 25.425, *p* = 0.000, *η^2^* = 0.204], with significantly higher accuracy observed in the high motor skill group than in the low motor skill group.

A significant main effect of motor skills was observed [*F*_(1, 99)_ = 19.109, *p* = 0.000, *η^2^* = 0.162], indicating significantly higher accuracy in the high motor skill group than in the low motor skill group. However, the main effect of memory difficulty was nonsignificant [*F*_(1, 99)_ = 3.349, *p* = 0.070, *η^2^* = 0.033].

### 3.3. fNIRS results

**Figure 3:**
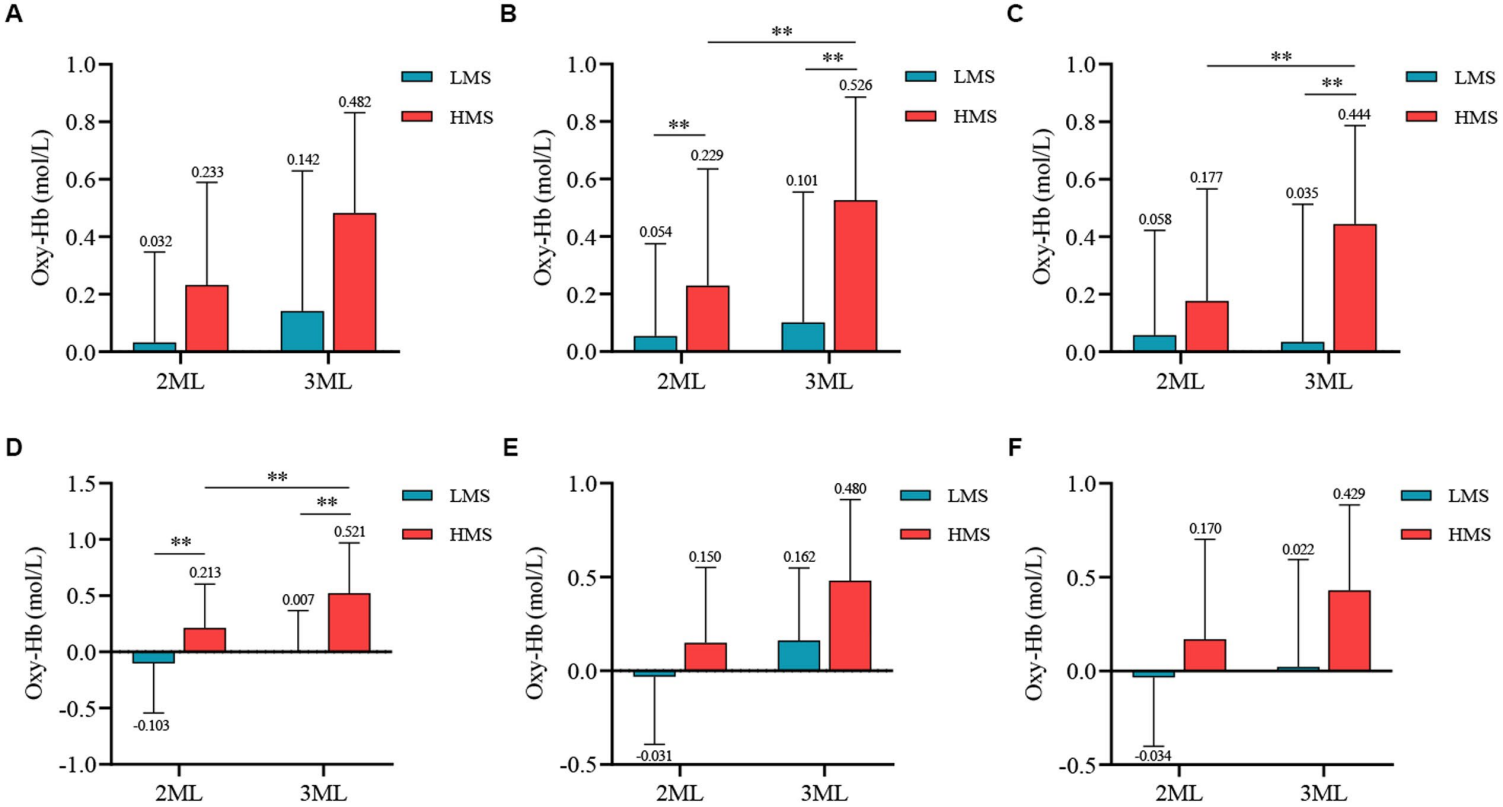
Differences in Oxy-Hb concentration between different motor skill groups and memory difficulties. ^**^p < 0.01; **(A)** The R-DLPFC result, **(B)** The L-DLPFC result, **(C)** The R-PTBA result, **(D)** The L-PTBA result, **(E)** The R-FPA result, and **(F)** The L-FPA result; 2MU, two-memory load; 3MU, three-memory load; LMS, Low motor skill group; and HMS, High motor skill group.

**Figure 4:**
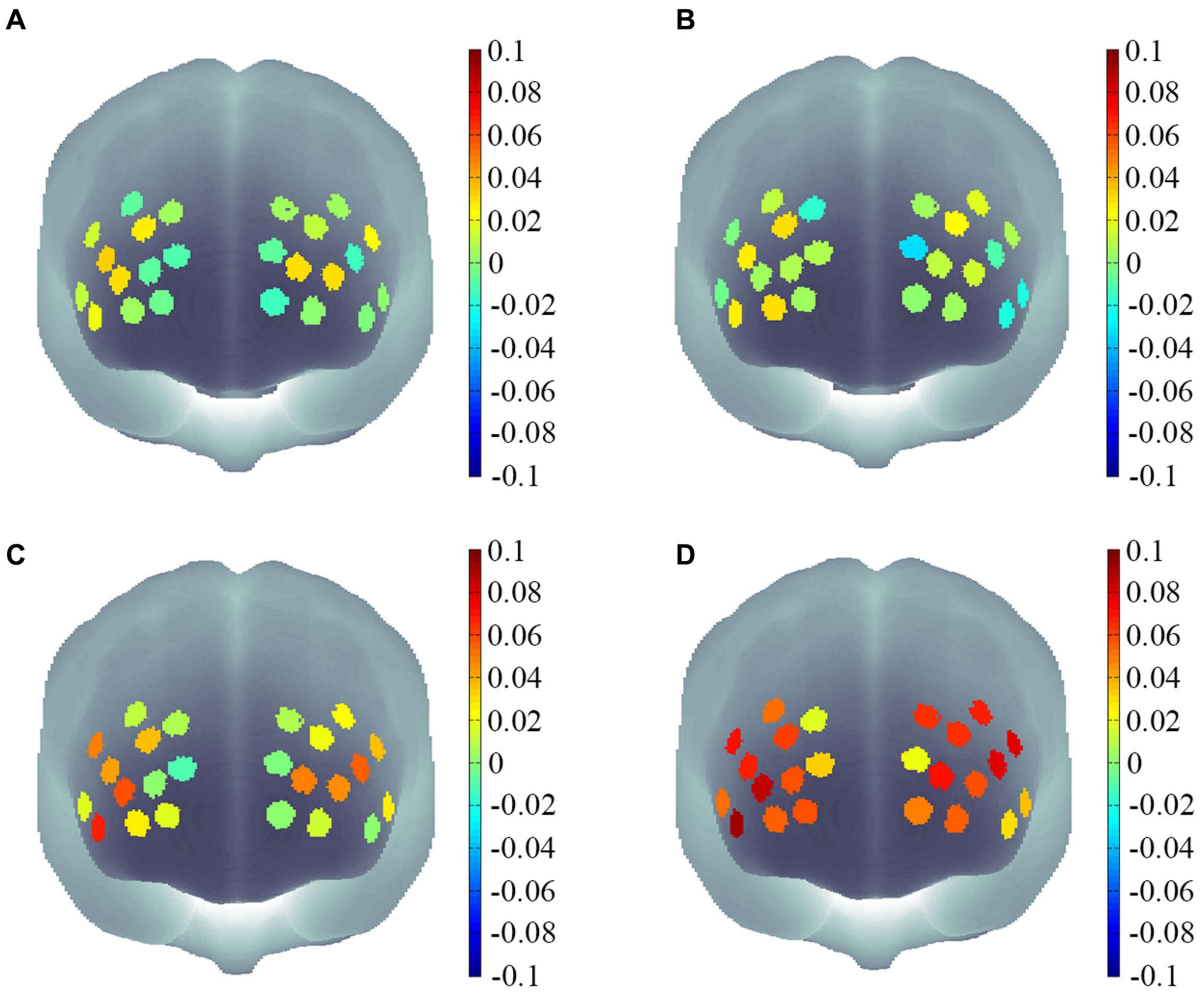
Channel activation maps for different motor skill groups under different memory difficulties. **(A)** Channel activation levels for the low motor skill group under two-memory load conditions. **(B)** Channel activation levels for the low motor skill group under three-memory load conditions. **(C)** Channel activation levels for the high motor skill group under two-memory load conditions. **(D)** Channel activation levels for the high motor skill group under three-memory load conditions.

The participants were divided into high and low-motor skill groups by using the median split method. A 2 (Motor Skill: High, Low) × 2 (Memory Difficulty: 2 Memory Load, 3 Memory Load) mixed-factor ANOVA was conducted to statistically analyze the PFC Oxy-Hb concentration for different groups under different working memory task loads (Figures 3, 4).

#### 3.3.1. Right dorsolateral prefrontal cortex

The interaction effect between motor skills and memory difficulty was nonsignificant [*F*_(1, 99)_ = 2.334, *p* = 0.130, *η^2^* = 0.023]. The main effect of motor skills was significant [*F*_(1, 99)_ = 19.857, *p* = 0.000, *η^2^* = 0.167], indicating that the high motor skill group exhibited significantly higher Oxy-Hb concentrations during the memory task than the low motor skill group. The main effect of memory difficulty was also significant [*F*_(1, 99)_ = 15.380, *p* = 0.000, *η^2^* = 0.134], with significantly higher Oxy-Hb concentrations observed during the three-memory load condition than during the two-memory load condition.

#### 3.3.2. Left dorsolateral prefrontal cortex

A significant interaction effect was observed between motor skills and memory difficulty [*F*_(1, 99)_ = 11.436, *p* = 0.001, *η^2^* = 0.104]. The simple effects analysis revealed that a significant main effect of memory difficulty was observed within the high motor skill group [*F*_(1, 99)_ = 35.473, *p* = 0.000, *η^2^* = 0.264], with significantly higher Oxy-Hb concentrations observed during the three-memory load condition than during the two-memory load condition. Under the two-memory load condition, the main effect of motor skills was significant [*F*_(1, 99)_ = 5.641, *p* = 0.019, *η^2^* = 0.054], indicating significantly higher Oxy-Hb concentrations in the high motor skill group than in the low motor skill group. Similarly, under the three-memory load condition, the main effect of motor skills was significant [*F*_(1, 99)_ = 27.665, *p* = 0.000, *η^2^* = 0.218], with the high motor skill group exhibiting significantly higher Oxy-Hb concentrations than the low motor skill group.

The main effect of motor skills was significant [*F*_(1, 99)_ = 19.480, *p* = 0.000, *η^2^* = 0.164], indicating significantly higher Oxy-Hb concentrations during the task in the high motor skill group than in the low motor skill group. The main effect of memory difficulty was also significant [*F*_(1, 99)_ = 21.690, *p* = 0.000, *η^2^* = 0.180], with significantly higher Oxy-Hb concentrations observed during the three-memory load condition than during the two-memory load condition.

#### 3.3.3. Right triangular part of the Broca’s area

A significant interaction effect was observed between motor skills and memory difficulty [*F*_(1, 99)_ = 11.985, *p* = 0.001, *η^2^* = 0.108]. The simple effects analysis revealed that the main effect of memory difficulty was significant within the high motor skill group [*F*_(1, 99)_ = 22.230, *p* = 0.000, *η^2^* = 0.183], with significantly higher Oxy-Hb concentrations observed during the three-memory load condition than during the two-memory load condition. Under the three-memory load condition, a significant main effect of motor skills was observed [*F*_(1, 99)_ = 24.963, *p* = 0.000, *η^2^* = 0.201], indicating significantly higher Oxy-Hb concentrations in the high motor skill group than in the low motor skill group.

A significant main effect of motor skills was observed [*F*_(1, 99)_ = 15.642, *p* = 0.000, *η^2^* = 0.136], indicating that the high motor skill group had significantly higher Oxy-Hb concentrations during the task than the low motor skill group. The main effect of memory difficulty was also significant [*F*_(1, 99)_ = 8.421, *p* = 0.005, *η^2^* = 0.078], with significantly higher Oxy-Hb concentrations observed during the three-memory load condition than during the two-memory load condition.

#### 3.3.4. Left triangular part of the Broca’s area

The interaction effect between motor skills and memory difficulty was significant [*F*_(1, 99)_ = 5.119, *p* = 0.026, *η^2^* = 0.049]. The simple effects analysis revealed that a significant main effect of memory difficulty was observed within the high motor skill group [*F*_(1, 99)_ = 26.985, *p* = 0.000, *η^2^* = 0.214], with significantly higher Oxy-Hb concentrations observed during the three-memory load condition than during the two-memory load condition. Under the two-memory load condition, the main effect of motor skills was also significant [*F*_(1, 99)_ = 14.600, *p* = 0.000, *η^2^* = 0.129], indicating that the high motor skill group had significantly higher Oxy-Hb concentrations than the low motor skill group. Similarly, under the three-memory load condition, a significant main effect of motor skills was observed [*F*_(1, 99)_ = 39.417, *p* = 0.000, *η^2^* = 0.285], with significantly higher Oxy-Hb concentrations in the high motor skill group than in the low motor skill group.

The main effect of motor skills was significant [*F*_(1, 99)_ = 35.697, *p* = 0.000, *η^2^* = 0.265], indicating significantly higher Oxy-Hb concentrations during the task in the high motor skill group than in the low motor skill group. The main effect of memory difficulty was also significant [*F*_(1, 99)_ = 22.553, *p* = 0.000, *η^2^* = 0.186], with significantly higher Oxy-Hb concentrations observed during the three-memory load condition than during the two-memory load condition.

#### 3.3.5. Right frontopolar area

The interaction effect between motor skills and memory difficulty was nonsignificant [*F*_(1, 99)_ = 2.332, *p* = 0.130, *η^2^* = 0.023]. However, a significant main effect of motor skills was observed [*F*_(1, 99)_ = 14.525, *p* = 0.000, *η^2^* = 0.128], indicating that the high motor skill group had significantly higher Oxy-Hb concentrations during the task than the low motor skill group. Additionally, the main effect of memory difficulty was significant [*F*_(1, 99)_ = 33.705, *p* = 0.000, *η^2^* = 0.254], with significantly higher Oxy-Hb concentrations observed during the three-memory load condition than during the two-memory load condition.

#### 3.3.6. Left frontopolar area

The interaction effect between motor skills and memory difficulty was nonsignificant [*F*_(1, 99)_ = 3.110, *p* = 0.081, *η^2^* = 0.030]. However, the main effect of motor skills was significant [*F*_(1, 99)_ = 15.084, *p* = 0.000, *η^2^* = 0.132], indicating that the high motor skill group exhibited significantly higher Oxy-Hb concentrations during the task than the low motor skill group. Additionally, the main effect of memory difficulty was significant [*F*_(1, 99)_ = 7.520, *p* = 0.007, *η^2^* = 0.071], with significantly higher Oxy-Hb concentrations observed during the three-memory load condition than during the two-memory load condition.

## 4. Discussion

Early childhood is a crucial period for developing motor skills and cognition (Stodden et al., 2008). Early motor skill development serves as a critical foundation for achieving higher levels of motor skill proficiency in later life. Rapid brain development in early childhood enables rapid cognitive development, with 3–6 years considered a critical age for brain development in young children. During this stage, brain development allows for cognitive advancement (Huttenlocher, 2002; Xiongtao et al., 2019; Hilderley et al., 2023). Motor development is constrained by the coordination and control abilities of the brain (Wade and Whiting, 1986), and working memory performance has a close relationship with neural development. Adolescents with higher motor development levels demonstrate superior working memory performance (Ludyga et al., 2018). Based on these findings, this study explored the relationship between motor development and working memory performance in children of very young age. The study results confirmed the association between motor development and both behavioral accuracy in working memory tasks and Oxy-Hb concentrations in the PFC, thereby providing empirical evidence that motor development is related to working memory performance in early childhood.

### 4.1. Influence of motor skills on behavioral performance in working memory tasks

Children aged 10–12 years showed that motor skills are related to reaction time in the Sternberg Memory Test, with children having higher motor skills exhibiting faster task response times (Ludyga et al., 2020). In another study involving children aged 7–10 years, a correlation was observed between motor ability and accuracy in working memory tasks, but no correlation was observed with reaction time (Lin et al., 2023). These findings are consistent with the present study results. Additionally, the interaction effect between motor skills and working memory demonstrated that the high motor skill group exhibited significantly higher accuracy than the low motor skill group, regardless of memory difficulty. Furthermore, the low motor skill group exhibited a significant decrease in accuracy with an increase in memory difficulty. Other studies have reported that motor skills are significantly correlated with reaction time in adolescents aged 10–15 years, but no significant correlation was observed with accuracy (Ludyga et al., 2018). Visuospatial working memory contributes to children’s motor learning (Bo and Seidler, 2009; Seidler et al., 2012; Buszard et al., 2016; Jongbloed-Pereboom et al., 2019), as the storage, encoding, and reproduction of motor processes rely on cognitive processing. Understanding the test rules and testing of motor skills are closely intertwined with working memory processes (Bo et al., 2011; Wozniak et al., 2022). The decision-making and selection steps involved in executing actions are intertwined with executive functions, including working memory (Yang and Wang, 2018). According to the dynamic system perspective, continuous interactions among the brain, body, and ever-changing environment occur during cognitive development (Gottwald et al., 2016). This possibly indicates that the environment synchronously influences both memory and motor development. These perspectives support the higher accuracy noted in the high motor skill group. In a study, university students exhibited a decreasing trend in behavioral accuracy when working memory difficulty increased (Lou et al., 2019). This agrees with the decreased accuracy in the low motor skill group under increased task load in this study. However, even under increased task load, the high motor skill group maintained higher accuracy, confirming the higher stability of memory accuracy in this group. Furthermore, the study results showed no differences in reaction time, which may be because of the young age of the participants, as they may not yet completely understand the relationship between accuracy and reaction time.

### 4.2. Impact of motor skills on Oxy-Hb concentration in the ROI

An interaction effect was observed between motor skills and the Oxy-Hb concentration in the left dorsolateral prefrontal cortex (L-DLPFC), right triangular part of the Broca’s area (R-PTBA), and left triangular part of the Broca’s area (L-PTBA) brain regions during working memory tasks. This indicates that individuals with high motor skills have a higher brain activation level in these regions during working memory tasks. ERP studies conducted in children (age: 10–15 years) have reported that compared with children with low motor skills, those with high motor skills exhibited higher P3 amplitudes during working memory tasks (Ludyga et al., 2018). This result has also been confirmed by studies involving children of age 7–10 years (Lin et al., 2023). In children around the age of 6 years, tennis practice could effectively enhance the blood oxygen efficiency in the left PFC during working memory tasks, as observed through increased Oxy-Hb concentrations in this region (Lai et al., 2020). These findings offer some support for the study results. Furthermore, this study confirms that even among children around 5 years of age, motor skills have an interactive influence on the brain oxygen efficiency during working memory tasks.

The PFC is a key brain region involved in working memory (Narayanan et al., 2005; Jia et al., 2022; Voegtle et al., 2022; Li et al., 2023). The DLPFC and ventrolateral prefrontal cortex (VLPFC) regions of the PFC exhibit linearly increasing activation during the maintenance phase of working memory tasks (Narayanan et al., 2005). Additionally, the hemodynamic response of the PFC, measured based on changes in oxyhemoglobin concentrations, is closely related to motor learning (Ono et al., 2015). Under both memory load conditions, the high motor skill group demonstrated significantly higher Oxy-Hb concentrations in the PFC, particularly in the L-DLPFC and L-PTBA regions, than the low motor skill group. This suggests that children with high motor skills can quickly recruit neural resources and focus them on the memory task, irrespective of the working memory load. The results also revealed that during the three-memory load condition, the high motor skill group had significantly higher Oxy-Hb concentrations in the PFC, specifically in the L-DLPFC, R-PTBA, and L-PTBA regions, than during the two-memory load condition. This indicates that the high motor skill group has higher sensitivity to increases in task difficulty and can engage additional activation in these relevant brain regions to complete the memory task. By contrast, the low motor skill group did not show this difference. Furthermore, increased demands for movement-related motor coordination have a more significant and specific impact on working memory in children and adolescents (Ludyga et al., 2022). The PFC is involved in the motor sequence learning process (Polskaia et al., 2023). Motor learning and execution rely on neural circuits forming the PFC (Chambon et al., 2011). Coordinative demands and non-automated bodily activities lead to the activation of the same brain regions responsible for controlling higher-order cognitive processes (Anguera et al., 2010; Diamond and Ling, 2016; Yang and Wang, 2018). Therefore, motor actions with cognitive challenges, such as procedural skills and motor transitions, may stimulate cognitive function development (Lin et al., 2023). This possibly explains why better activation in the working memory brain regions was observed in the high-scoring group in the MOBAK test.

Working memory contributes to motor learning. Individual differences in the working memory capacity possibly indicate the differences in attention control during movement, thereby affecting the tendency for conscious engagement in motor performance (Buszard et al., 2016). In Duijn’s EEG study, visuospatial working memory capacity was positively correlated with the coherence of activation between T8 and Fz (regions associated with visuospatial working memory and motor planning) during a hockey passing task. Individuals with a higher visuospatial working memory capacity mostly process motor tasks in a visuospatial manner (Duijn et al., 2017). Buszard’s study revealed that visuospatial working memory is related to implicit motor learning (which relies less on verbal memory for acquiring motor skills), which allows for stable performance even in complex environments (such as under pressure or when multitasking) (Buszard et al., 2016). According to the adaptive capacity model, the link between cognitive-demanding movements and brain development relies on the neurophysiological system’s ability to adapt to stimuli (Raichlen and Alexander, 2017). In other words, the brain–body relationship functions on a “use it or lose it” basis, where motor development requires higher-order cognition and cognitive development facilitates motor learning. This helps us comprehend how differences in motor skills among young children can impact their cognitive development process (Lin et al., 2023).

### 4.3. Limitations

In this study, a modified version of the delayed match task was used to measure working memory capacity (Luck and Vogel, 2013). Considering that the participants were 4–6-year-old children with limited cognitive abilities, they were only required to judge whether the test array differed from the sample array, based on the general spatial configuration. This means that the accuracy of the participants’ responses was only related to the overall spatial arrangement of the arrays. In other studies, researchers employed a more sensitive measure of working memory capacity, where the test array always changed, and participants had to indicate specific changes (Gold et al., 2006; Pedale et al., 2022). Therefore, it must be acknowledged that the task array used in this study may have lacked sensitivity in measuring working memory capacity. Future research could enhance the sensitivity of the task array to improve the quality of working memory capacity assessment.

## 5. Conclusion

This study confirms that motor development is closely related to working memory in early childhood. Children with high motor skills demonstrated higher accuracy in maintaining working memory while performing the task. Additionally, these children with high motor skills displayed greater activation in PFC regions for different memory difficulties. These findings have practical implications for improving and promoting motor and cognitive development in young children.

## Data availability statement

The original contributions presented in the study are included in the article/Supplementary material, further inquiries can be directed to the corresponding author.

## Ethics statement

The studies involving humans were approved by Ethics Committee of Shaanxi Normal University. The studies were conducted in accordance with the local legislation and institutional requirements. Written informed consent for participation in this study was provided by the participants’ legal guardians/next of kin.

## Author contributions

QZ: Data curation, Software, Visualization, Writing – original draft, Writing – review & editing. AC: Conceptualization, Formal analysis, Methodology, Writing – review & editing. BS: Investigation, Resources, Writing – review & editing. YW: Data curation, Methodology, Writing – review & editing. QM: Investigation, Resources, Writing – review & editing. FZ: Resources, Writing – review & editing. XG: Resources, Writing – review & editing. MZ: Resources, Writing – review & editing. BL: Investigation, Resources, Writing – review & editing. KN: Funding acquisition, Methodology, Writing – review & editing.

## References

[ref2] Anguera J. A. Reuter-Lorenz P. A. Willingham D. T. Seidler R. D. J. J. C. N. (2010). Contributions of spatial working memory to visuomotor learning. J. Cogn. Neurosci. 22, 1917–1930. doi: 10.1162/jocn.2009.21351, PMID: 19803691

[ref3] Barnett L. M. Stodden D. Cohen K. E. Smith J. J. Lubans D. R. Lenoir M.. (2016). Fundamental movement skills: an important focus. J. Teach. Phys. Educ. 35, 219–225. doi: 10.1123/jtpe.2014-0209

[ref4] Blankenship T. L. Keith K. Calkins S. D. Bell M. A. (2018). Behavioral performance and neural areas associated with memory processes contribute to math and reading achievement in 6-year-old children. Cogn. Dev. 45, 141–151. doi: 10.1016/j.cogdev.2017.07.002, PMID: 29861542 PMC5978002

[ref5] Bo J. S. Jennett R. D. Research, S. J. E. B (2011). Working memory capacity correlates with implicit serial reaction time task performance. Exp. Brain Res. 214, 73–81. doi: 10.1007/s00221-011-2807-8, PMID: 21809082

[ref6] Bo J. Seidler R. D. (2009). Visuospatial working memory capacity predicts the organization of acquired explicit motor sequences. J. Neurophysiol. 101, 3116–3125. doi: 10.1152/jn.00006.2009, PMID: 19357338 PMC2694099

[ref7] Buszard T. Farrow D. Zhu F. F. Masters R. S. W. (2016). The relationship between working memory capacity and cortical activity during performance of a novel motor task. Psychol. Sport Exerc. 22, 247–254. doi: 10.1016/j.psychsport.2015.07.005

[ref8] Carruthers P. (2013). Evolution of working memory. Proc. Natl. Acad. Sci. U. S. A. 110, 10371–10378. doi: 10.1073/pnas.1301195110 23754428 PMC3690618

[ref9] Chambon V. Domenech P. Pacherie E. Koechlin E. Baraduc P. Farrer C. (2011). What are they up to? The role of sensory evidence and prior knowledge in action understanding. PLoS One 6:e17133. doi: 10.1371/journal.pone.0017133 21364992 PMC3041795

[ref10] Constantinidis C. Klingberg T. (2016). The neuroscience of working memory capacity and training. Nat. Rev. Neurosci. 17, 438–449. doi: 10.1038/nrn.2016.43 27225070

[ref11] Diamond A. (2000). Close interrelation of motor development and cognitive development and of the cerebellum and prefrontal cortex. Child Dev. 71, 44–56. doi: 10.1111/1467-8624.00117, PMID: 10836557

[ref12] Diamond A. Ling D. S. J. D. C. N. (2016). Conclusions about interventions, programs, and approaches for improving executive functions that appear justified and those that, despite much hype, do not. Dev. Cogn. Neurosci. 18, 34–48. doi: 10.1016/j.dcn.2015.11.005, PMID: 26749076 PMC5108631

[ref13] Duijn T. V. Buszard T. Hoskens M. C. J. Masters R. S. W. (2017). Discerning measures of conscious brain processes associated with superior early motor performance: capacity, coactivation, and character. Prog. Brain Res. 234, 245–261. doi: 10.1016/bs.pbr.2017.06.013, PMID: 29031466

[ref14] Gold J. M. Fuller R. L. Robinson B. M. Mcmahon R. P. Braun E. L. Luck S. J. (2006). Intact attentional control of working memory encoding in schizophrenia. J. Abnorm. Psychol. 115, 658–673. doi: 10.1037/0021-843X.115.4.658, PMID: 17100524

[ref15] Gottwald J. M. Achermann S. Marciszko C. Lindskog M. Gredeback G. (2016). An embodied account of early executive-function development: prospective motor control in infancy is related to inhibition and working memory. Psychol. Sci. 27, 1600–1610. doi: 10.1177/0956797616667447, PMID: 27765900 PMC5154392

[ref16] Herrmann C. Ferrari I. Wlti M. Wacker S. Kühnis J. (2018). MOBAK-KG testmanual (english). Basic motor competencies in kindergarten.

[ref17] Hilderley A. J. Wright F. V. Taylor M. J. Chen J. L. Fehlings D. (2023). Functional neuroplasticity and motor skill change following gross motor interventions for children with diplegic cerebral palsy. Neurorehabil. Neural Repair 37, 16–26. doi: 10.1177/15459683221143503, PMID: 36524254 PMC9896542

[ref18] Huttenlocher P. R. (2002). Neural plasticity: The effects of environment on the development of the cerebral cortex. Cambridge: Harvard University Press.

[ref19] Iacobucci D. Posavac S. S. Kardes F. R. Schneider M. J. Popovich D. L. (2015). Toward a more nuanced understanding of the statistical properties of a median split. J. Consum. Psychol. 25, 652–665. doi: 10.1016/j.jcps.2014.12.002

[ref20] Jia W. Shengnan Z. Tiaotiao L. Xuyuan Z. Xin T. Wenwen B. (2022). Directional prefrontal-thalamic information flow is selectively required during spatial working memory retrieval. Front. Neurosci. 16:1055986. doi: 10.3389/fnins.2022.1055986, PMID: 36507330 PMC9726760

[ref21] Jongbloed-Pereboom M. Nijhuis-van der Sanden M. W. G. Steenbergen B. (2019). Explicit and implicit motor sequence learning in children and adults; the role of age and visual working memory. Hum. Mov. Sci. 64, 1–11. doi: 10.1016/j.humov.2018.12.007, PMID: 30639705

[ref22] Lai Y. Wang Z. Yue G. H. Jiang C. (2020). Determining whether tennis benefits the updating function in young children: a functional near-infrared spectroscopy study. Appl. Sci. Basel 10:407. doi: 10.3390/app10010407

[ref23] Li K. Yang J. Becker B. Li X. (2023). Functional near-infrared spectroscopy neurofeedback of dorsolateral prefrontal cortex enhances human spatial working memory. Neurophotonics 10:025011. doi: 10.1117/1.NPh.10.2.025011, PMID: 37275655 PMC10234406

[ref24] Lin C. C. Hsieh S. S. Huang C. J. Kao S. C. Chang Y. K. Hung T.-M. (2023). The unique contribution of motor ability to visuospatial working memory in school-age children: evidence from event-related potentials. Psychophysiology 60:e14182. doi: 10.1111/psyp.14182, PMID: 36094017 PMC10078500

[ref25] Lindenberger U. Li S. C. Gruber W. Mueller V. (2009). Brains swinging in concert: cortical phase synchronization while playing guitar. BMC Neurosci. 10, 1–12. doi: 10.1186/1471-2202-10-22, PMID: 19292892 PMC2662862

[ref26] Lou Y. Zhou C. L. Lu Y. Z. (2019). Study on characteristics of biological motion working memory processing of college students with different physical activity levels. J. Cap. Sport. Univ. 31, 364–384. doi: 10.14036/j.cnki.cn11-4513.2019.04.015

[ref27] Luck S. J. Vogel E. K. (2013). Visual working memory capacity: from psychophysics and neurobiology to individual differences. Trends Cogn. Sci. 17, 391–400. doi: 10.1016/j.tics.2013.06.006, PMID: 23850263 PMC3729738

[ref28] Ludyga S. Gerber M. Kamijo K. (2022). Exercise types and working memory components during development. Trends Cogn. Sci. 26, 191–203. doi: 10.1016/j.tics.2021.12.004, PMID: 35031211

[ref29] Ludyga S. Herrmann C. Mücke M. Andrä C. Brand S. Pühse U.. (2018). Contingent negative variation and working memory maintenance in adolescents with low and high motor competencies. Neural Plast. 2018, 1–9. doi: 10.1155/2018/9628787, PMID: 29849576 PMC5932462

[ref30] Ludyga S. Mücke M. Kamijo K. Andrä C. Pühse U. Gerber M.. (2020). The role of motor competences in predicting working memory maintenance and preparatory processing. Child Dev. 91, 799–813. doi: 10.1111/cdev.13227, PMID: 30791099

[ref31] McKay C. A. Shing Y. L. Rafetseder E. Wijeakumar S. (2021). Home assessment of visual working memory in pre-schoolers reveals associations between behaviour, brain activation and parent reports of life stress. Dev. Sci. 24:e13094. doi: 10.1111/desc.13094, PMID: 33523548

[ref32] Narayanan N. S. Prabhakaran V. Bunge S. A. Christoff K. Fine E. M. Gabrieli J. D. E. J. N. (2005). The role of the prefrontal cortex in the maintenance of verbal working memory: an event-related FMRI analysis. Neuropsychology 19, 223–232. doi: 10.1037/0894-4105.19.2.223, PMID: 15769206

[ref33] Ono Y. Noah J. A. Zhang X. Nomoto Y. Suzuki T. Shimada S.. (2015). Motor learning and modulation of prefrontal cortex: an fNIRS assessment. J. Neural Eng. 12:066004. doi: 10.1088/1741-2560/12/6/066004, PMID: 26401727

[ref34] Pedale T. Mastroberardino S. del Gatto C. Capurso M. Bellagamba F. Addessi E.. (2022). Searching for a relationship between early breastfeeding and cognitive development of attention and working memory capacity. Brain Sci. 13:53. doi: 10.3390/brainsci13010053, PMID: 36672035 PMC9856597

[ref35] Piazza C. Bacchetta A. Crippa A. Mauri M. Grazioli S. Reni G.. (2019). “Preprocessing pipeline for fNIRS data in children” in *Paper presented at the 15th Mediterranean Conference on Medical and Biological Engineering and Computing (MEDICON)*. UNESCO World Heritage Univ, Coimbra, Portugal.

[ref36] Polskaia N. St-Amant G. Fraser S. Lajoie Y. (2023). Involvement of the prefrontal cortex in motor sequence learning: a functional near-infrared spectroscopy (fNIRS) study. Brain Cogn. 166:105940. doi: 10.1016/j.bandc.2022.105940, PMID: 36621187

[ref37] Quilez-Robres A. Moyano N. Cortes-Pascual A. (2021). Task monitoring and working memory as executive components predictive of general and specific academic achievements in 6-9-year-old children. Int. J. Environ. Res. Public Health 18:6681. doi: 10.3390/ijerph18136681, PMID: 34206172 PMC8295744

[ref38] Raichlen D. A., and, Alexander G. E., (2017). Adaptive capacity: an evolutionary neuroscience model linking exercise, cognition, and brain health. Trends Neurosci. 40, 408–421. doi: 10.1016/j.tins.2017.05.001, PMID: 28610948 PMC5926798

[ref39] Seidler R. D. Bo J. Anguera J. A. (2012). Neurocognitive contributions to motor skill learning: the role of working memory. J. Mot. Behav. 44, 445–453. doi: 10.1080/00222895.2012.672348, PMID: 23237467 PMC3534841

[ref40] Simmering V. R. (2012). The development of visual working memory capacity during early childhood. J. Exp. Child Psychol. 111, 695–707. doi: 10.1016/j.jecp.2011.10.007, PMID: 22099167

[ref41] Stodden D. F. Goodway J. D. Langendorfer S. J. Roberton M. A. Rudisill M. E. Garcia C.. (2008). A developmental perspective on the role of motor skill competence in physical activity: an emergent relationship. Quest 60, 290–306. doi: 10.1080/00336297.2008.10483582

[ref42] Strangman G. Culver J. P. Thompson J. H. Boas D. A. J. N. (2002). A quantitative comparison of simultaneous BOLD fMRI and NIRS recordings during functional brain activation. NeuroImage 17, 719–731. doi: 10.1006/nimg.2002.1227, PMID: 12377147

[ref1] Voegtle A. Reichert C. Hinrichs H. Sweeney-Reed C. M. (2022). Repetitive anodal TDCS to the frontal cortex increases the P300 during working memory processing. Brain Sci. 12:1545. doi: 10.3390/brainsci12111545, PMID: 36421869 PMC9688092

[ref43] Wade M. G. Whiting H. T. A. (1986). Motor Development in Children: Aspects of Coordination and Control. The Netherlands: Martinus Nijhoff

[ref44] Wozniak M. Schmidt T. T. Wu Y.-H. Blankenburg F. Hohwy J. (2022). Differences in working memory coding of biological motion attributed to oneself and others. Hum. Brain Mapp. 43, 3721–3734. doi: 10.1002/hbm.25879, PMID: 35466500 PMC9294297

[ref45] Wu S. Qian C. Shen X. Zhang X. Huang Y. Chen S.. (2022). Spike prediction on primary motor cortex from medial prefrontal cortex during task learning. J. Neural Eng. 19:046025. doi: 10.1088/1741-2552/ac8180, PMID: 35839739

[ref46] Wu Z. J. Wang Z. Y. Wang Q. (2021). The neuroscience of motor development: the future path and layout. Sci. China Life Sci. 51, 619–633. doi: 10.1360/SSV-2020-0242

[ref47] Xiang M. Q. Huang W. Q. Li W. J. Liu S. F. Liao B. G. (2022). The application of functional near-infrared spectroscopy in exercise-cognitive neuroscience. Sci. Tech. Guide Newspaper 40, 89–96. doi: 10.3981/j.issn.1000-7857.2022.10.009

[ref48] Xie S. Wu D. Yang J. Luo J. Chang C. Li H. (2021). An fNIRS examination of executive function in bilingual young children. Int. J. Biling. 25, 516–530. doi: 10.1177/1367006920952881

[ref49] Xiongtao D. Pantelis H. Jane-Ling W. Deoni S. C. L. Hans-Georg M. (2019). Longitudinal associations between white matter maturation and cognitive development across early childhood. Hum. Brain Mapp. 40, 4130–4145. doi: 10.1002/hbm.24690, PMID: 31187920 PMC6771612

[ref50] Yang H. Y. Wang S. M. (2018). Neurophysiological mechanisms of motor learning. J. Wuhan Inst. Phys. Educ. 52, 85–89. doi: 10.15930/j.cnki.wtxb.2018.08.014

[ref51] Yifan C. Yanglan Y. Ruoyu N. Ying L. (2018). Selective effects of postural control on spatial vs. nonspatial working memory: a functional near-infrared spectral imaging study. Front. Hum. Neurosci. 12:243. doi: 10.3389/fnhum.2018.00243, PMID: 29950981 PMC6008320

